# Coproducing Knowledge of the Implementation of Complex Digital Health Interventions for Adults with Acquired Brain Injury and their Communication Partners: Protocol for a Mixed Methods Study

**DOI:** 10.2196/35080

**Published:** 2022-01-10

**Authors:** Melissa Miao, Emma Power, Rachael Rietdijk, Deborah Debono, Melissa Brunner, Alexander Salomon, Ben Mcculloch, Meg Rebecca Wright, Monica Welsh, Bastian Tremblay, Caleb Rixon, Liz Williams, Rosemary Morrow, Jean-Christophe Evain, Leanne Togher

**Affiliations:** 1 University of Technology Sydney Sydney Australia; 2 University of Sydney Sydney Australia; 3 See Authors' Contributions Australia; 4 Brain Injury Rehabilitation Unit South Australian Brain Injury Rehabilitation Service Hampstead Rehabilitation Centre Adelaide Australia; 5 Genyus Network Melbourne Australia; 6 Brain Injury Rehabilitation Community and Home South Australian Brain Injury Rehabilitation Service Hampstead Rehabilitation Centre Adelaide Australia; 7 Acquired Brain Injury Rehabilitation Centre Caulfield Hospital Alfred Health Network Melbourne Australia

**Keywords:** priority setting, public involvement, implementation science, internet interventions, acquired brain injury, delivery of health care, caregivers, speech-language pathology, brain injury, mobile phone

## Abstract

**Background:**

The Social Brain Toolkit, conceived and developed in partnership with stakeholders, is a novel suite of web-based communication interventions for people with brain injury and their communication partners. To support effective implementation, the developers of the Social Brain Toolkit have collaborated with people with brain injury, communication partners, clinicians, and individuals with digital health implementation experience to coproduce new implementation knowledge. In recognition of the equal value of experiential and academic knowledge, both types of knowledge are included in this study protocol, with input from stakeholder coauthors.

**Objective:**

This study aims to collaborate with stakeholders to prioritize theoretically based implementation targets for the Social Brain Toolkit, understand the nature of these priorities, and develop targeted implementation strategies to address these priorities, in order to support the Social Brain Toolkit’s implementation.

**Methods:**

Theoretically underpinned by the Nonadoption, Abandonment, Scale-up, Spread, and Sustainability (NASSS) framework of digital health implementation, a maximum variation sample (N=35) of stakeholders coproduced knowledge of the implementation of the Social Brain Toolkit. People with brain injury (n=10), communication partners (n=11), and clinicians (n=5) participated in an initial web-based prioritization survey based on the NASSS framework. Survey completion was facilitated by plain English explanations and accessible captioned videos developed through 3 rounds of piloting. A speech-language pathologist also assisted stakeholders with brain injury to participate in the survey via video teleconference. Participants subsequently elaborated on their identified priorities via 7 web-based focus groups, in which researchers and stakeholders exchanged stakeholder perspectives and research evidence from a concurrent systematic review. Stakeholders were supported to engage in focus groups through the use of visual supports and plain English explanations. Additionally, individuals with experience in digital health implementation (n=9) responded to the prioritization survey questions via individual interview. The results will be deductively analyzed in relation to the NASSS framework in a coauthorship process with people with brain injury, communication partners, and clinicians.

**Results:**

Ethical approval was received from the University of Technology Sydney Health and Medical Research Ethics Committee (ETH20-5466) on December 15, 2020. Data were collected from April 13 to November 18, 2021. Data analysis is currently underway, with results expected for publication in mid-2022.

**Conclusions:**

In this study, researchers supported individuals with living experience of acquired brain injury, of communicating with or clinically supporting someone post injury, and of digital health implementation, to directly access and leverage the latest implementation research evidence and theory. With this support, stakeholders were able to prioritize implementation research targets, develop targeted implementation solutions, and coauthor and publish new implementation findings. The results will be used to optimize the implementation of 3 real-world, evidence-based interventions and thus improve the outcomes of people with brain injury and their communication partners.

**International Registered Report Identifier (IRRID):**

DERR1-10.2196/35080

## Introduction

### Terminology and Style

Coproduction of knowledge is a “process whereby professionals and those traditionally on the receiving end of their ‘expertise’ (eg, patients/service users/marginalized citizens) can collaborate with the goal of achieving outcomes that arguably cannot be achieved otherwise” [1]. This requires a shift in power [2,3] from the narrow profession of academia to the broader public, underpinned by an epistemological shift that values knowledge derived from experience as much as knowledge derived through research [1,3]. This “epistemic pluralism” is internationally espoused in the United Nations Educational, Scientific and Cultural Organization (UNESCO) Recommendation on Open Science [4] and underpins this study of the implementation of digital health interventions for adults with acquired brain injury (ABI) and their communication partners (eg, family and friends). To reflect this pluralism, this study protocol uses a nontraditional writing style in which the direct comments of coauthors with living experience of ABI, and of communicating with or clinically supporting someone with ABI, have been interleaved with traditional academic writing. This approach allows for the sharing of collective insights into research methods and conveys the equal importance of both experiential and academic knowledge.

Additionally, this protocol refers to the “living experience” of stakeholders, quoted in the present tense, as an equally valued commentary on the research methods. As author CR explains from their living experience of ABI:

It is imperative to use vocabulary that empowers and increases the agency of a person whose life has been impacted by a brain injury, identifying with the tense in which they are experiencing their journey. One's language carries great weight, and with that, a responsibility to not wound, constrict or hold someone hostage to a traditionally perceived narrative of recovery. As such, using “living experience” can reflect the wisdom and expertise an individual possesses, and continues to build, as they move along their life journey.

### Background

ABI, such as stroke or traumatic brain injury (TBI), commonly causes cognitive communication disorders, in which impairments in underlying cognitive skills lead to difficulties with communication [5]. These communication disorders can introduce challenges for a person’s social participation and relationships [6], employment [7,8], and mental health [9], while the ABI remains a “hidden” or “invisible” disability [10]. Author BM describes from their living experience:

If my TBI were to be a characterized in a word, it would be “isolation.” People with brain injury often appear superficially “fine” with no head wounds or disfigurement, whereas in reality I lost the majority of my personality and capacity to relate to people for over half a decade.

Communities and close others of people with ABI also experience psychosocial, health, and economic burdens as a result of ABI [11-13]. The communication styles and skills of these close others can positively or negatively affect the communication skills of people with ABI [14-16]. Author BT observes from their living experience as a communication partner of someone with ABI:

I found myself out of my depth and without the tools needed to be able to help my friend and communicate with him in such a way that would not add to his mental health issues relating to his new life with an ABI. I had to train myself to not say things to him that would emphasize that an error had been made due to his ABI. The medical, mental health and rehabilitation care is rightly all focused on the patient. However, when that person is moving towards coming home again, the carers that will be living on a day-to-day basis with the person with an ABI are not given the same level of support. A lot of the time during the months my friend was in hospital and rehabilitation could have been spent educating me on how I could best serve my friend, and a number of the mistakes I have made could have been avoided.

Therefore, communication partner training (CPT) to assist individuals interacting with people with ABI is considered best practice in the management of communication disorders following ABI [17-19].

The current and future need for CPT exceeds global health care capacity to provide communication rehabilitation through qualified speech-language pathologists [20,21]. More than 135 million people worldwide currently live with an ABI [20], and this number is projected to grow continually [20,22]. To address this challenge of scale and equity in health care access, a novel suite of digital health interventions known as the “Social Brain Toolkit” [23] has been designed to enable adults with ABI, their close others and communities to access web-based communication training. The Social Brain Toolkit is being developed in Australia and will be available internationally.

The Social Brain Toolkit contains 3 interventions which leverage digital health functionalities and encompass established principles of CPT. First, “convers-ABI-lity” offers a web-based conversation skills training program for adults with ABI and their familiar communication partners (eg, family, partners, and friends). convers-ABI-lity converts the existing efficacious CPT programs of TBI Express [24] and TBIconneCT [25,26] into a bespoke all-in-one platform of self-directed web-based training and telehealth sessions with a speech-language pathologist. Second, “interact-ABI-lity” offers self-directed web-based CPT for individuals interacting with people with ABI, including paid support workers and the public. Finally, “social-ABI-lity” provides self-directed web-based social media training for people with ABI, to increase social connections for individuals who may have limited opportunity for interactions with communication partners. It aims to enhance social participation, social communication skills, and a sense of self or identity postinjury [27].

Digital health interventions such as the Social Brain Toolkit face many implementation challenges, including technological adaptability and complexity [28], cost [28,29], workflow impact [30], and long-term sustainability [29,31]. Addressing these complexities requires dialogue with potential end users early in the process of intervention design [32,33]. In addition, there is an ethical imperative for stakeholder involvement in the conduct of health care research [34,35]. The inclusion of “societal actors” in the research process has been enshrined internationally in the UNESCO Recommendation on Open Science [4] and the national Statement for Community and Consumer Involvement in Health Care Research in Australia [36]. The coproduction of research may facilitate research translation [36] and reduce research waste by ensuring that the research undertaken generates meaningful results for end users [37,38]. However, research systems and processes may be ill-equipped to support such collaboration [1,3,39]. Coproducing knowledge within these structures can be an extremely resource-intensive and politically and ethically fraught task [3,40], even without the additional complexity introduced by cognitive or communication impairments, such as those associated with ABI. Therefore, sharing of methodological knowledge and guidance on how to undertake this endeavor most effectively [37,38] is important to support future coproduction of research with people with ABI, their communication partners, and clinicians.

### Aims

In this study, researchers, together with people with ABI, their communication partners, and clinicians, as well as individuals with living experience of digital health implementation, aim to coproduce an understanding of the implementation of the Social Brain Toolkit, to enable these interventions to reach their intended users and meet stakeholder needs in a feasible, scalable, sustainable, and acceptable manner. Specifically, this study has the following aims: (1) to obtain stakeholder prioritization of theoretically based implementation targets for the Social Brain Toolkit, (2) to understand stakeholder perspectives of these priorities, and (3) to collaborate with stakeholders to identify implementation strategies targeting these priorities.

## Methods

### Ethics

This research was ethically approved by the University of Technology Sydney (UTS) Health and Medical Research Ethics Committee (ETH20-5466) on December 15, 2020. Recruitment flyers included an invitation to email the researcher to express interest in the project. Communication partners, clinicians and industry or research experts were emailed a link to complete electronic participant information and consent forms. Participants with ABI were invited to provide informed written consent using accessible, plain English participant information and consent forms that were adapted to incorporate visual supports and explained via video call by a qualified speech-language pathologist using supported communication strategies [41]. Screening for the capacity to consent is described in the inclusion criteria (the screening protocol is also included as Multimedia Appendix 1) [42]. Participant demographic information is reported as an aggregate to preserve participant anonymity.

People with ABI and communication partners were reimbursed for their participation at the annual hourly rate recommended by Health Consumers New South Wales (NSW) [43]. To support the implementation of the Social Brain Toolkit, its developers sought to learn from the expert living experience of people with ABI, their communication partners and clinicians, as well as those who have implemented digital health interventions. From this paradigm, the individual experiential knowledge of these health care stakeholders is seen as valuable for the implementation of digital health interventions and can be valued by respecting, seeking, recording, and sharing it in academic publications, acknowledging this contribution publicly and personally, and financially reimbursing those who otherwise may not receive payment for this knowledge [38]. This reimbursement also aimed to minimize any undue burden of research participation. Potential participants were advised of this arrangement in the participant information form to facilitate decision-making around any potential economic burden of participation. This financial reimbursement was optional, and in some cases declined, with reasons including altruism and potential ineligibility for benefit schemes if any payment was received. The researchers respected these wishes. Of interest, there were procedural barriers to providing participants with direct payment via the university payroll, which would have granted institutional affiliation as equals. These barriers included a lack of precedence, university requirements for participants to complete several modules of mandatory web-based induction training as staff, and taxation requirements. Therefore, researchers were requested to provide payment via electronic gift cards and could only maximize participant autonomy with their payments by providing a choice of retailer for the gift card. This may be seen as another example of a systemic structure within academia that is not readily suited to research coproduction. Author CR reflects from their living experience of both ABI and research participation that:

Providing an option for an expert to choose between cash or voucher not only creates more equality; it also increases their individual agency, self-confidence and self-worth via their perceived value from the researchers, and most importantly pushes back against the narrative of being a consumer, and reduces their sense of being a burden but instead someone bringing significant value.

Finally, all participants received a certificate of appreciation for their participation, and for some participants, this was anecdotally expressed as the most meaningful acknowledgment.

As part of the ethical considerations of the study, participants were advised of the risks of emotional distress during participation and had opportunities to take breaks or seek support from the researchers or other psychological services. Emotional support was required by one participant, requiring vigilance and counseling skills within the small group format, which was aided by the clinical background of the facilitators. Author BM highlights that a focus group may be a participant’s first encounter with another person with ABI. Individuals with living experience may therefore feel a range of emotions associated with their participation. For example, BM recalls it was “sad to see the variety of different stories of so many different people” who had this “shared devastation.” However, BM believes this was also inversely “decentralizing” in a positive way, as such a group “provokes a feeling of unity” that we were “all here for the same thing.”

### Design

**Figure 1:**
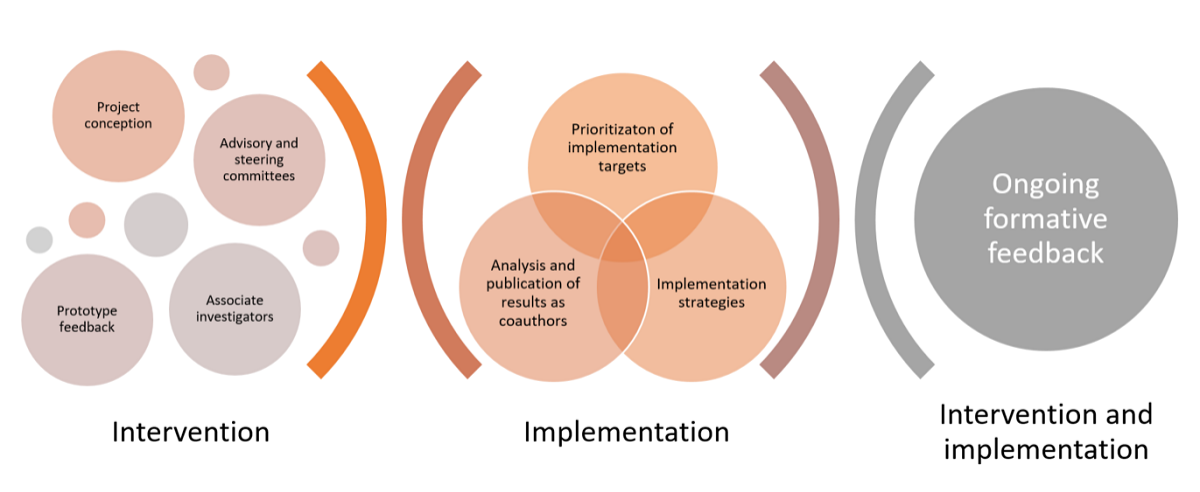
Stakeholder involvement during the development of the Social Brain Toolkit.

The coproduction of implementation knowledge in this study is part of a broader effort to involve stakeholders throughout the development of the Social Brain Toolkit (Figure 1). The development of the Social Brain Toolkit is led by author LT, a speech-language pathologist, clinical researcher, and Director of the Acquired Brain Injury Communication Lab [23] in Sydney, Australia, together with research team members, speech-language pathologists and authors EP, RR, MB, and MM, in collaboration with the technology vendor Changineers [44]. The project was jointly conceived and reviewed by stakeholders including people with ABI and their communication partners, community partners such as Brain Injury Australia, and funding partner icare NSW. This was a requirement for funding at project inception, and subsequent funding for this study and related studies [45]. These stakeholders have continued to participate in advisory and steering committees for the project, provided feedback on early prototypes, and will provide ongoing formative feedback on the interventions and their implementation [45]. Clinicians delivering convers-ABI-lity were included as associate investigators in the project. People with living experience of ABI, of being a communication partner, or of clinically supporting someone with ABI, are coauthors of this study protocol and will be included in the coauthorship of its results.

Theoretically underpinned by the Nonadoption, Abandonment, Scale-up, Spread, and Sustainability (NASSS) theoretical framework for digital health implementation [46,47] in both analysis and data collection protocols, this mixed methods study gives voice to the priorities and perspectives of 2 distinct cohorts: (1) a purposive, maximum variation sample of people with ABI, communication partners, and clinicians; and (2) a purposive sample of individuals with experience implementing digital health interventions for any health condition. The use of a complexity-based theoretical framework such as the NASSS [46] enabled the consideration and integration of the needs of multiple stakeholders, including clinician end users who will implement the intervention in complex, adaptive health care systems. People with ABI, communication partners, and clinicians participated in (1) an initial prioritization survey and (2) subsequent focus groups exploring these priorities. Individuals with experience in digital health implementation responded to the prioritization survey via individual interviews. Interview methods were used due to both limited participant availability and a need to balance homogeneity and heterogeneity in the focus groups, particularly in relation to power and differences and similarity of experience [48], as participants with experience implementing digital health were not required to have clinical or research experience in ABI specifically. The results of this group were analyzed separately to understand and compare these perspectives with those of potential end users.

### Participants

#### Inclusion and Exclusion Criteria

All participants had to (1) be older than 18 years and (2) have adequate English proficiency to participate in the study without the aid of an interpreter. 

Participants with ABI were self-identified and could participate if they met the following criteria:

1. Had adequate capacity to consent to participate in the study. The capacity to consent was ascertained during a video call with a qualified speech-language pathologist according to our adapted consenting process protocol (Multimedia Appendix 1) [42], which includes relevant questions adapted from the University of California Brief Assessment of Capacity to Consent [42]. People with ABI unable to respond adequately to all 5 questions presented using supported communication strategies would be excluded from the study.
2. Were discharged from hospital.
3. Were ≥6 months post injury.
4. Were based in Australia.

The exclusion criterion for participants with ABI was a self-reported mild ABI or concussion in which minimal or no observable or self-reported changes in social communication function were present.

Communication partners of a person with ABI were self-identified individuals who (1) interacted at least once a week with a person with ABI, (2) had not sustained an ABI themselves, and (3) were based in Australia.

Recruitment of participants with ABI and communication partners was focused on participants based in Australia to reflect the Australian development context of the Social Brain Toolkit and because the available forms of reimbursement were only usable within Australia.

Clinicians were self-identified (1) as qualified and currently practicing allied health professionals (2) with a caseload of which at least 20% included people with ABI.

Research or industry experts in digital health implementation were required to have (1) a published, peer-reviewed academic track record in English concerning digital health implementation or delivery for any health condition or (2) a leadership position in digital health delivery in industry or the health care system, or (3) both.

There were no restrictions on any other factors (eg, gender or level of clinical experience), and maximum variation in these factors was preferred where possible.

#### Sampling

To obtain a purposive, maximum variation sample of people with ABI, communication partners, clinicians, and individuals with experience in digital health implementation, we distributed recruitment flyers tailored to each group via relevant organizational and researcher social media channels, organizational websites, and email distribution lists. Individuals with experience in digital health implementation were emailed directly through publicly listed contact information, such as web-based university researcher profiles or researcher networks.

The final sample (N=35) included people with living experience of ABI, of being a communication partner of people with ABI, of clinically supporting people with ABI, of implementing digital health, or a combination of these experiences (Table 1). Proportionally, the perspectives of people with ABI and their communication partners were prioritized by recruiting twice the number of health care stakeholders compared with clinicians, and by obtaining industry and research perspectives separately.

The authors of this study protocol include participants with living experience of ABI, of being a communication partner of a person with ABI, or of providing clinical care for people with ABI. Participants with ABI, communication partners and clinicians will also have the opportunity to participate as coauthors in the analysis, interpretation and write-up of the study findings.

**Table 1:** Participant demographic information reported as an aggregate to preserve participant anonymity (N=35).

	Adults with experience of ABI^a^ (n=10)	Communication partners (n=11)	Clinicians (n=5)	Individuals with experience of digital health implementation (n=9)
Living experience	9 with living experience of ABI 1 with living experience of both ABI and clinically supporting people with ABI	11 with living experience of being a communication partner of someone with ABI	5 with clinical experience supporting people with ABI as speech-language pathologists	8 with living experience implementing digital health interventions for any condition 1 with living experience of both ABI and digital health implementation
Sex	7 male 3 female	4 male 7 female	1 male 4 female	6 male 3 female
Location	Australia (as per inclusion criteria)	Australia (as per inclusion criteria)	1 from the United Kingdom 1 from the Netherlands 3 from Australia	1 from Denmark 9 from Australia
Education	1 with a graduate diploma or certificate 4 with a bachelor’s degree 4 with a certificate or diploma 1 with a high school certificate	1 with a PhD 1 with a master’s degree 2 with a graduate diploma or certificate 3 with a bachelor’s degree 3 with a certificate or diploma 1 with a high school certificate	2 with a master’s degree 2 with a graduate diploma or certificate 2 with a bachelor’s degree	9 with a PhD
Age (years)	2 aged 25-34 4 aged 35-44 2 aged 45-54 1 aged 55-64 1 aged >65	1 aged 25-34 2 aged 35-44 2 aged 45-54 1 aged 55-64 5 aged >65	1 aged 18-24 2 aged 25-34 1 aged 35-44 1 aged 45-54	N/A^b^
Time postinjury	9 participants >12 months 1 participant <12 months	N/A	N/A	N/A
Clinical experience	N/A	N/A	3 working for >10 years with 80%-100% caseload with ABI 1 working for <5 years with 80%-100% caseload with ABI 1 working for <5 years with 20% caseload with ABI	N/A
Relationship to person with ABI	N/A	3 friends 3 partners 5 family members	N/A	N/A

^a^ABI: acquired brain injury.

^b^N/A: not applicable.

### Data Collection

#### Overview

People with ABI, communication partners, and clinicians each participated in (1) an initial prioritization survey and (2) subsequent focus groups discussing priorities identified in the initial surveys. Individuals with experience in digital health implementation participated in individual interviews that addressed the same initial prioritization questions. The following section reports data collection procedures, rationales, and reflections on these procedures from participant coauthors AS, MRW, LW, MW, and BM.

#### Surveys

Surveys included basic demographic questions, a Likert scale, an open-ended qualitative question for each domain of the NASSS framework, and an overall prioritization ranking of all domains (Multimedia Appendix 2). The inclusion of Likert scales and free-text responses was intended to capture the strength and nature of feeling participants may or may not have about each of the ranked items, enabling the complexity of the rankings to be understood, and if required, reflected in the time allocation of focus groups.

To enable stakeholders to access existing academic knowledge of digital health implementation [49], the survey was theoretically underpinned by the NASSS framework of digital health implementation [46] and underwent multiple rounds of piloting before administration:

1. An initial version of the survey was developed from a NASSS-based interview schedule [50], as applied to the Social Brain Toolkit [45]. This first version was piloted via a live audience poll during an in-person oral presentation of each NASSS domain to a range of funding and health care stakeholders.
2. On the basis of this pilot, a second version of the survey was developed, incorporating asynchronous video explanations of each domain, including large captions, slowed speech rate, and plain English. This version was piloted with 4 members of the research team (EP, RR, DD, and LT).
3. The video script and phrasing of the survey questions were further refined into a third version piloted with a speech-language pathologist with clinical experience in ABI, an informal carer from a culturally and linguistically diverse (CALD) background, and a usability engineer.
4. Their input was incorporated into the fourth and final version delivered to participants, in which videos and questions were made more concise, and survey usability was refined.

The final survey questions, including video transcripts, are included in Multimedia Appendix 2. Supporting videos explaining each question are available in Multimedia Appendices 3-9. Author MRW, who has living experience of ABI, notes “the video clips that were provided meant we were able to watch it repeatedly to get our ideas out.” Clinician coauthor MW notes that the adaptations made to ensure the accessibility of surveys and videos reflects “the critical nature of these supports in enabling participants with unique needs secondary to ABI to be involved in this research.” For participants with ABI, this survey was administered by a speech-language pathologist (MM) via video teleconference, using supported communication techniques [41].

#### Focus Groups

In the focus groups, stakeholders examined the top 4 stakeholder priorities from the NASSS framework [46,47], obtained via the prioritization survey ranking (Multimedia Appendix 2). The domains 1-4 received the highest scores from stakeholders. Therefore, domains 5-7 were excluded from further investigation, as they received the lowest scores.

A deductive analysis [51] of the qualitative survey data revealed a significant overlap between participants’ discussion of domains 1 and 2 of the NASSS framework [46]. Therefore, focus group discussions for these 2 domains were combined, followed by a discussion targeting domain 4, and a discussion focused on domain 3 (see Multimedia Appendix 10 for a detailed time allocation).

On the basis of stakeholder priorities, the following plain English questions were posed to participants:

1. Domain 1: Who can use the Social Brain Toolkit (1) straightaway, (2) with support, or (3) would be unable to use it? How can we help and what supports can we provide?
2. Domain 2: Which device (ie, smartphone, tablet, desktop computer) would you prefer to use and why? How can we help/what supports can we provide to use the technology?
3. Domain 3: What is the value or benefit of the Social Brain Toolkit? Who should pay for the Tools? Who would you trust to tell you about the Tools (look online, a therapist or service, people with brain injury, research, organizations)?
4. Domain 4: How can we help the Social Brain Toolkit fit into your routine (ie, (1) doing a course by yourself, (2) doing homework, and (3) online video calls and appointments)?

Stakeholders with ABI, communication partners, and clinicians explored these prioritized questions during the focus groups. To coproduce knowledge of each of the above priority domains, author MM synthesized and presented the stakeholders with (1) relevant preliminary research findings from a concurrent systematic review underpinned by the NASSS framework [52] and (2) relevant qualitative data from the prioritization survey, based on deductive content analysis [51] using the NASSS framework [46] (Multimedia Appendix 10). All information was presented using visual supports and scaffolds via Microsoft PowerPoint (Microsoft Corporation) in plain English using supported communication techniques [41]. Author BM notes from living experience that the “acknowledgement and unhurried language” of these communication techniques allows “for more candid, useful answers.” Author MRW also reflects “the slide meant you were able to understand what the study was all about.”

The focus group method was selected as it allows participants to share and compare their experiences [48]. This rationale was corroborated by the experience of author AS, who reflects that:

Participating as a member of a [focus] group (1) stimulated my thinking, (2) encouraged me to make a contribution to the discussion, (3) helped me evaluate my ideas by listening to other group members’ responses to my suggestions, (4) provided me with immediate feedback and (5) helped build up my confidence.

For author AS, the benefits of participating in a focus group were that it “enabled me to appreciate some of challenges faced by others” and “to think more deeply about those challenges.” Similarly, author MRW believes the “chance to listen to others’ experiences and thoughts” was beneficial. Author LW, who has clinical experience supporting people with ABI and their families, similarly reflects on the benefits of sharing in a focus group and hearing others’ perspectives, while also feeling that it was important to “stay in the background so that those with living experience could do the core part of the talking and contributing given their expertise.” This reflects the varied impact of difference or similarity of experience in focus groups [48] and may require proactive management by facilitators.

As focus groups require rapport among participants [48], time was allocated for personal introductions (Multimedia Appendix 10). For the groups that also met for collective discussions, author BM reflects that a full introduction of every participant could have contextualized each person’s contribution in plenary discussions. Author AS reflects on the importance of this for disclosure in focus groups [48]:

This was the first time I had met the other participants. This lack of familiarity with fellow participants could have inhibited my contribution because I wasn’t confident the others would interpret my contribution in the spirit it was intended.

Author AS believes that a potential opportunity to informally meet other participants beforehand may have facilitated discussion in the focus group itself.

It is important to note that introductions were and are not straightforward for participants with ABI. Author BM highlights from their living experience that talking about an ABI can be very personal and difficult:

Depending on the personality and stage of recovery, I'd liken talking to a clinician about your TBI to something like...delineating to a stranger how you lost your virginity!

This reflects the power imbalance that may affect disclosure [48] between a clinician and an individual with ABI. Therefore, BM advises the following:

To generate clearer, more candid answers from people with TBI, the researchers or clinicians are best served acknowledging the delicacy of the situation and reiterating how intense the gravity of a TBI is/was.

Researchers also sought to convey this respect by advising participants that they were able to disclose as much or as little information as they felt comfortable, and researchers did not probe for details that were not volunteered.

Each focus group was 3 hours in duration. Author MRW notes that this required participants “setting time aside to do it, but it’s like that with everything.” Each hour included a break to mitigate fatigue. Author BM notes from their living experience, “It’s usually an appreciated acknowledgement that working memory and attention spans are scarce with a TBI.” Each hour of the focus groups included (1) a synthesis of survey and systematic review data for 1-2 domains and (2) small group discussions (see Multimedia Appendix 10 for a detailed time allocation). In author MRW’s experience, “giving us something to concentrate on over a period of time meant we were able to think about it a lot more and get it sorted out in our own heads.”

Focus groups were arranged based on participant availability to bring together as many participants as possible at a common time to share ideas. Participants received reminders, instructions, and an outline of the focus groups before participation. Author BM reflects that “follow up phone calls, gentle reminders” and “consistency and thoughtful language” were all “very much appreciated.” There were a total of 7 focus groups (n=26) containing 3 to 6 stakeholders, each facilitated by 1 to 2 members of the research team. Author BM notes that “breaking the groups up to ensure there weren't too many contributions and noise at the one time” was helpful for participants with ABI, and author AS highlights the benefit of “having a group facilitator to assist.” As data were collected and recorded using web-based video call software, researchers trained participants in how to use the software’s mute and camera options. As video call “hosts,” the researchers also familiarized themselves with the mute and camera functions to preserve participant privacy during scheduled breaks in the calls if needed.

Participant input from the first focus group (n=3) was incorporated into the information presented to a larger, subsequent session of 4 focus groups (n=17), where each focus group’s insights were also recounted in plenary discussions in between the small group sessions (Multimedia Appendix 10). These findings were, in turn, shared for discussion with the 2 final groups (n=3 and n=3, respectively). This enabled sharing of insights among focus groups, in addition to the dialogue within each group, and the wider dialogue between researchers and stakeholders.

Data collection occurred entirely on the web via secure videoconferencing on Microsoft Teams (Microsoft Corporation). Participants with ABI had each used the software before during their initial screening, and all participants were provided with visually supported initial instructions on how to use the functions of the video call software. All participants were advised that they could log onto the call early to troubleshoot technology if needed. All participants were connected for the full duration of the focus groups without technical difficulty, with the exception of 1 participant whose participation was scheduled over 2 sessions owing to low battery. The focus groups were audio and video recorded using the built-in recording functions of the videoconferencing platform.

#### Interviews

Interviews with individuals with research or industry experience of digital health implementation used the same prioritization questions as the surveys (Multimedia Appendix 2) to enable discussion of multiple issues within a limited timeframe. Interviews were conducted individually and ranged from 1 to 2.5 hours in duration based on participant availability. Data collection was also iterative, with insights from prior focus groups and interviews included as conversation prompts in subsequent interviews. Data collection occurred entirely on the web, with interviews conducted via secure videoconferencing on Microsoft Teams and audio and video recorded using the built-in recording functions.

### Analysis

#### Quantitative

Prioritization rankings, Likert scales, and demographic information from initial surveys were analyzed descriptively using Qualtrics (Qualtrics International) survey software.

#### Qualitative

Free-text survey responses were deductively analyzed [51] based on the NASSS framework [46] to enable qualitative data specific to stakeholder priorities to be extracted and shared in subsequent focus groups. All focus groups and interviews will be transcribed verbatim. To synthesize qualitative data and test the domains of the framework, author MM will deductively code interview data [51] against the NASSS framework [46] and lead an identical analysis of focus group data using a web-based codebook that will include the accessible videos, defining each domain to be coded (Multimedia Appendices 3-9). Deductive coding will be managed using Microsoft Excel 365 (Microsoft Corporation).

### Rigor

This protocol details stakeholder prioritization according to the REPRISE (Reporting Guideline for Priority Setting of Health Research) guidelines for reporting research priority setting [38]. All interview and focus group participants were given an opportunity to add to their original contributions verbally or in writing for inclusion as original data. All focus group transcripts will be verified against the original audio or video recordings by at least one person originally present in that focus group. All interview and focus group participants will be given an opportunity to member-check preliminary interpretations of their input. A second author will verify a random 25% of the total codes from focus groups and interviews using the aforementioned codebook. For focus group data, this verification will be conducted by a coauthor who was present in that focus group as either a researcher or participant. Any discrepancies will be resolved via consensus discussion.

### Evaluation

Stakeholder priorities and implementation strategies are directly informing the implementation of the Social Brain Toolkit. The outcomes of the implementation strategies will be investigated in a hybrid type 2 implementation–effectiveness study [53] of all 3 interventions in the Social Brain Toolkit [45].

### Publication

Stakeholders with living experience contributed to, critically reviewed, and are therefore listed as coauthors in the publication of this study protocol. They are also coauthors in the analysis, interpretation, and publication of the study results. MM conceptualized and formalized this coauthorship process in the grant proposals for this study. MM is facilitating this collaboration by email, telephone, video call, and Microsoft Word (Microsoft Corporation), according to each author’s communication preferences and accessibility requirements. In keeping with the UNESCO Recommendation on Open Science [4], researchers have obtained funding to enable both this study protocol and the forthcoming results of the contributions of participants to be available open access. Authors AS and MM highlight that journal requirements to include academic qualifications for the stakeholders coauthoring this protocol are artifacts of a system that prizes academic knowledge, which, although reported for transparency, may arguably be seen as less relevant than or even contradicting the value of living experience.

## Results

Australian National Health and Medical Research Council Postgraduate Scholarship funding was granted in November 2019. UTS Center for Carer’s research funding was granted in September 2020. icare NSW funding was received in November 2019. Ethical approval was received from the UTS Health and Medical Research Ethics Committee (ETH20-5466) on December 15, 2020.

Data were collected from April 13 to November 18, 2021. Data analysis is currently ongoing, with results expected for publication in mid-2022.

## Discussion

### Coproducing Implementation Knowledge

This implementation study combined (1) the experiential knowledge of people living with ABI, communicating with or clinically supporting people with ABI, and implementing digital health; with (2) academic knowledge in the form of current digital health implementation evidence and theory to (3) coproduce new implementation knowledge for 3 real-world interventions. As people with ABI, their communication partners, and clinicians are direct stakeholders in the development and implementation of digital health interventions targeting communication changes after ABI, there is an ethical imperative for their inclusion in intervention design, development, and delivery [35]. In the field of implementation science in particular, it is arguable that their input is essential for successful real-world implementation as early as intervention development [32] and while empirical data are still developing.

At the heart of this coproduction is a proactive exchange of power [2,3]. In the Social Brain Toolkit project as a whole, stakeholder involvement to date has included project conception, advisory input, feedback on prototypes, and the coproduction of implementation knowledge (Figure 1). In practice, this has required stakeholder input from multiple groups and individuals to varying degrees and at various times over the course of a multiyear program of research, commensurate with each stakeholder’s autonomy and availability. For instance, within the present implementation study, some stakeholders did not accept the offer for coauthorship, instead preferring to participate in surveys and focus groups as a one-off contribution, whereas others are investing ongoing time and effort to coauthor this study protocol and its eventual results. The power to decide one’s involvement in research can only begin when an opportunity is provided. Therefore, stakeholders can and should be given opportunities for involvement and contribution in research, which in some cases are opportunities that only researchers have the power to create or provide. For example, coauthorship of this study protocol required proactive invitation, inclusion, and facilitation by researchers, thus giving power to stakeholders to decide whether they may or may not want to be involved in this way.

When researchers seek to redress the imbalance of power in research, it can *empower* stakeholders. As author AS reflects, his involvement “provided me with the opportunity to make a contribution to a worthwhile project, and improve my self-esteem.” Author MRW reflects that:

Talking about my personal experience to someone who showed interest in my experiences - when some people might listen but won’t take it on board - that encourages you to talk about your own experiences.

Author MRW also reflects that “the encouragement that we got from [the researchers] and even other people with ABI and their communication partners was extremely encouraging.” Author BM says:

Getting a voice and having your contributions valued by people in impactful positions is liberating. Researchers trying to understand the details of the TBI journey is emboldening.

This protocol outlines the resource-intensive and politically and ethically challenging task of research coproduction [3,40] and how the consideration of cognitive or communication impairments, such as those associated with ABI, increased the complexity of the task. In this study, the facilitation of stakeholder involvement required proactive, premeditated support, including the investment of significant and ongoing time and effort to (1) guide stakeholders through the research process; (2) scaffold requests for input visually, verbally and in writing, according to individual communication preferences and accessibility requirements; (3) pilot and prepare resources in accessible, plain English with visual supports and supported communication techniques; and (4) secure grant funding in advance to reimburse participants for their expertise. The provision of these supports was necessary to affirm stakeholders’ value and potential for input at each stage of an otherwise unfamiliar research process. Although MM, EP, RR, MB, and LT’s backgrounds as speech-language pathologists and MM’s background training in accessibility aided the development of accessible supports for participants, the aforementioned interest, encouragement, support, and respect that was appreciated by stakeholders are arguably universal hallmarks of effective and respectful communication and collaboration, achievable by, and expected of [36], all researchers. Likewise, the additional time, effort, and funding invested are not unique to the speech-language pathology profession. This time-cost may be counterbalanced by the way that stakeholder prioritization enables the reduction of research waste [37], in this case by enabling a focus on implementation domains that were of most importance to potential end users.

In this study, there were numerous time points and methods by which stakeholders contributed to the implementation of the Social Brain Toolkit. In addition to prioritizing implementation research targets, stakeholders were involved in the development of targeted implementation strategies to address these priorities. A subgroup is currently involved in the interpretation of these findings for publication as coauthors. Additional opportunities for dialogue among stakeholders, and between stakeholders and researchers, were created by using collectively surveyed stakeholder priorities to inform focus groups, and cumulatively sharing each focus group’s data in subsequent focus groups and interviews. This collaborative dynamic was important to foster not only for successful focus groups [48] but moreover because the purpose of the research was to coproduce implementation knowledge. Researcher MM reflects that although common goals were initially outlined in individual surveys and interviews, this could also have been reiterated in subsequent focus groups. Although plenary discussions naturally led to discussions regarding the collaborative purpose of the focus groups, author BM recommends that researchers proactively facilitate a collaborative dynamic by (1) prefacing focus group discussion with a concise and jargon-free explanation of “this is what we’re doing, this is what your role is, and this why we’re doing it,” and (2) reminding participants “that the academic research goals are strictly aligned with the needs of TBI sufferers and their families, in that the research and collaboration is to build pathways that reduce problems and pain for TBI sufferers.”

This collaboration toward a common goal is at the core of research coproduction. However, in author MM’s experience, the time-limited nature of academic systems directly hinders research coproduction at every stage of research [1,3,39], as it inherently conflicts with the space and time required for the intensive and relational nature of effective collaboration. Yet stakeholder suggestions for the opportunity to build familiarity with other participants, and the need for researchers and clinical end users to discuss common goals clearly attest to this relational need.

### Strengths and Limitations

This study demonstrates potential methods of engaging stakeholders throughout the implementation research process, from prioritization to authorship. In particular, we share how to retain theoretical rigor in implementation science while capturing the highly nuanced and complex reality of implementation from multiple stakeholder perspectives. However, there are several limitations to this study in relation to stakeholder representation. First, there was a minority representation of stakeholders outside Australia. Although the inclusion of English-speaking users in Australia reflects the initial development context of the Social Brain Toolkit, future studies may seek to explore international and CALD perspectives. In addition, although this study included similar numbers of communication partners as people with ABI, author MM observed that communication partners were most reticent to participate, and therefore underrepresented, in the publication stages of research. A particularly proactive effort was and may be required to give voice to this population specifically. Finally, although we sought to recruit clinicians, managers, and policy makers, clinical participation was solely comprised of speech-language pathologists. Although they are conceivably the most likely clinician to recommend, deliver, or support the implementation of the Social Brain Toolkit, the perspective of other clinical professions, as well as health care managers and policy makers, could have contributed to a more multifaceted understanding of implementation from a clinical perspective.

### Conclusions

By making implementation theory directly accessible to multiple stakeholders and proactively facilitating a dialogue between stakeholder experience and implementation theory, we were able to coproduce new implementation knowledge to support the implementation of 3 real-world interventions, including targeted implementation strategies [45]. Author MW observes the following from a clinician perspective:

This paper, by its very nature, encourages reflection on the inaccessibility of research (both research processes and outcomes) to non-researchers and [this] work - towards breaking these barriers - is unlike anything I have seen before.

To our knowledge, this is the first published coauthorship of implementation research with people with living experience of ABI, of being a communication partner of someone with ABI, and of clinically supporting adults with ABI. As a person with living experience of ABI, author CR believes:

Embedding of the experiential wisdom of people with living experience builds a more robust evidence base and foundations of trust which are required to occupy a shared space for innovation.

Author CR concludes as follows:

[In this study, the researchers’] line of inquiry, pursuit of transparent feedback and desire for ongoing engagement in a relational, rather than transactional way, built a foundation of trust for how we might work together in cocreation. It is now for researchers to consume this new knowledge, reflect on it and act.

## Appendix

Multimedia Appendix 1 Consent screening protocol.

Multimedia Appendix 2 Prioritization survey.

Multimedia Appendix 3 Survey video for domain 1 of the NASSS as applied to the Social Brain Toolkit.

Multimedia Appendix 4 Survey video for domain 2 of the NASSS as applied to the Social Brain Toolkit.

Multimedia Appendix 5 Survey video for domain 3 of the NASSS as applied to the Social Brain Toolkit.

Multimedia Appendix 6 Survey video for Domain 4 of the NASSS as applied to the Social Brain Toolkit.

Multimedia Appendix 7 Survey video for Domain 5 of the NASSS as applied to the Social Brain Toolkit.

Multimedia Appendix 8 Survey video for Domain 6 of the NASSS as applied to the Social Brain Toolkit.

Multimedia Appendix 9 Survey video for Domain 7 of the NASSS as applied to the Social Brain Toolkit.

Multimedia Appendix 10 Focus group data collection.

## Abbreviations

ABI: acquired brain injury
CALD: culturally and linguistically diverse
CPT: communication partner training
NASSS: Nonadoption, Abandonment, Scale-up, Spread, and Sustainability
NSW: New South Wales
REPRISE: Reporting Guideline for Priority Setting of Health Research
TBI: traumatic brain injury
UNESCO: United Nations Educational, Scientific and Cultural Organization
UTS: University of Technology Sydney
