# Supplementary material for: Coproducing Knowledge of the Implementation of Complex Digital Health Interventions for Adults with Acquired Brain Injury and their Communication Partners: Protocol for a Mixed Methods Study
Source: JMIR Res Protoc. 2022 Jan 10;11(1):e35080. doi: 10.2196/35080 (PMC8787662; doi:10.2196/35080)
Supplement: Multimedia Appendix 2 [file resprot_v11i1e35080_app2.pdf]

Default Question Block

To start the survey, please enter your name.

First name

Surname

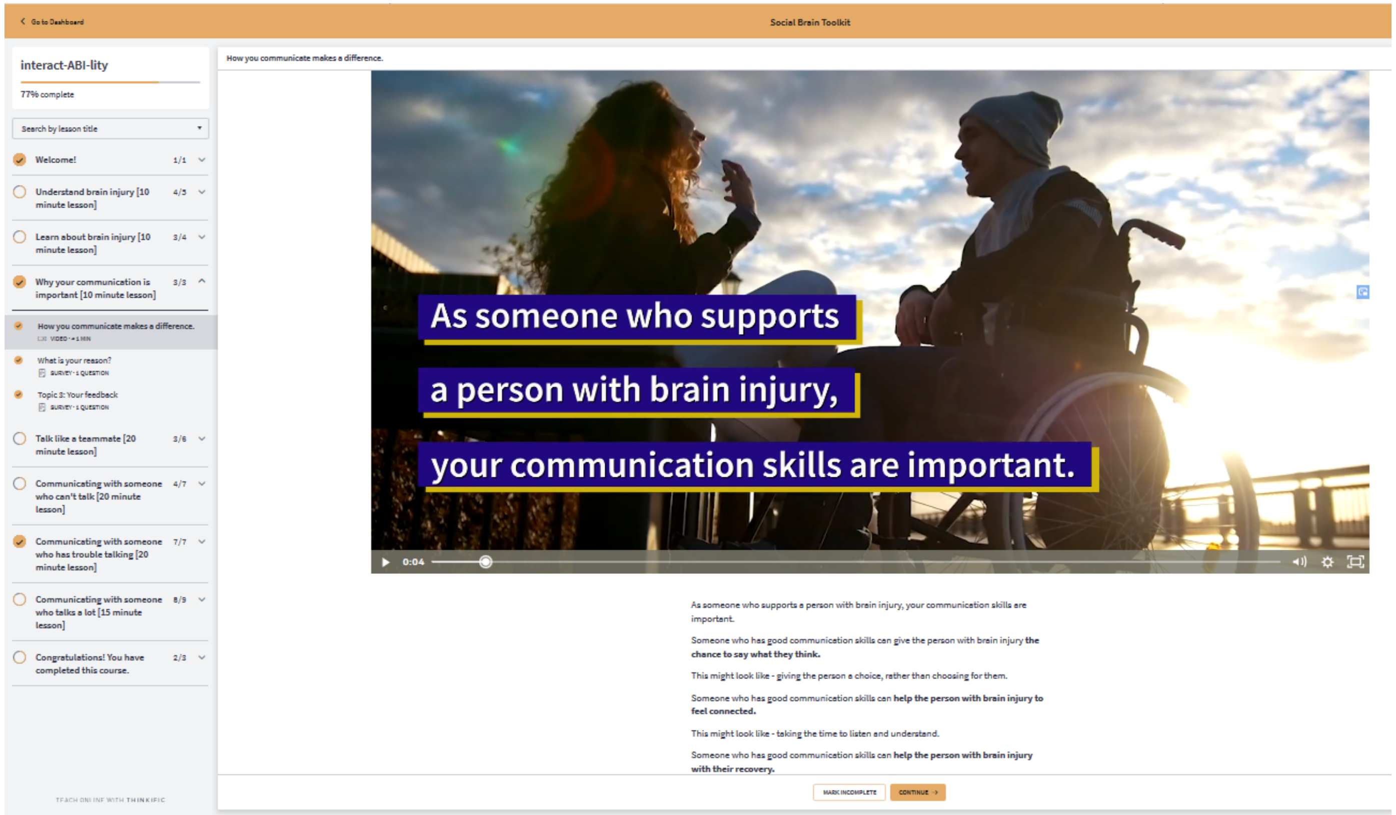

The Social Brain Toolkit is a collection of new **online communication training** tools:

- for people who have had a **brain injury**
- and the **people they communicate with** (e.g. family, friends, partners and paid support workers).

We will show you seven videos of **possible challenges of using the Social Brain Toolkit.**

We want to know **which ones you think are the most important**.

Challenge A: Difficulties related to brain injury

Another possible challenge is the difficulties related to brain injury. Here is a video explaining some reasons why.

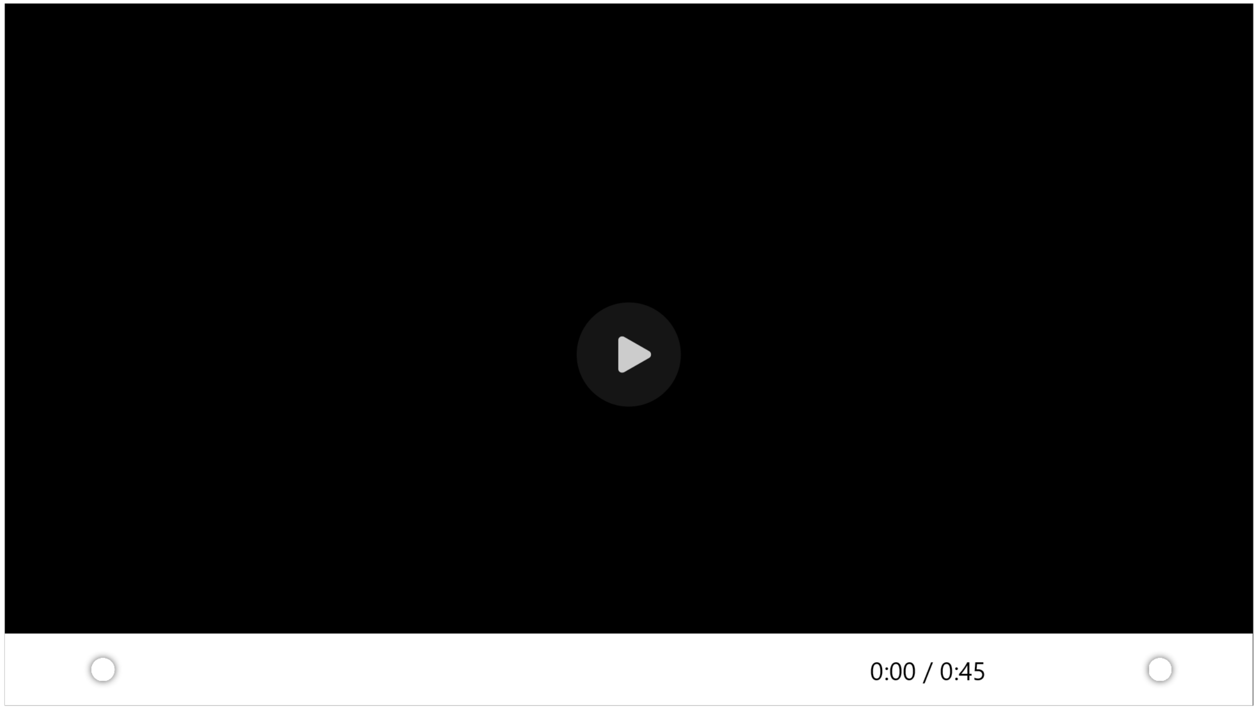

Here is a transcript of the video if you'd like to read it again.

The Social Brain Toolkit asks people to

- *learn online*
- *and sometimes talk online with a therapist.*

But brain injury can cause difficulties with

- *communication and*
- *memory*

Which can make it hard to:

- *remember content*
- *concentrate on content*
- *and communicate.*

Some people with brain injury sometimes have extra worries like:

- *damaged relationships.*
- *job loss*
- *domestic violence*
- *homelessness*

Which can make it hard to:

- *access the internet*
- *access healthcare*
- *practice together with someone online*

Difficulties related to brain injury will affect people's use of the Social

- Strongly agree
- Somewhat agree
- Neither agree nor disagree
- Somewhat disagree
- Strongly disagree

Why do you think so?

Challenge B: Using technology

A possible challenge of the Social Brain Toolkit is **using technology**.

Here is a video explaining some reasons why.

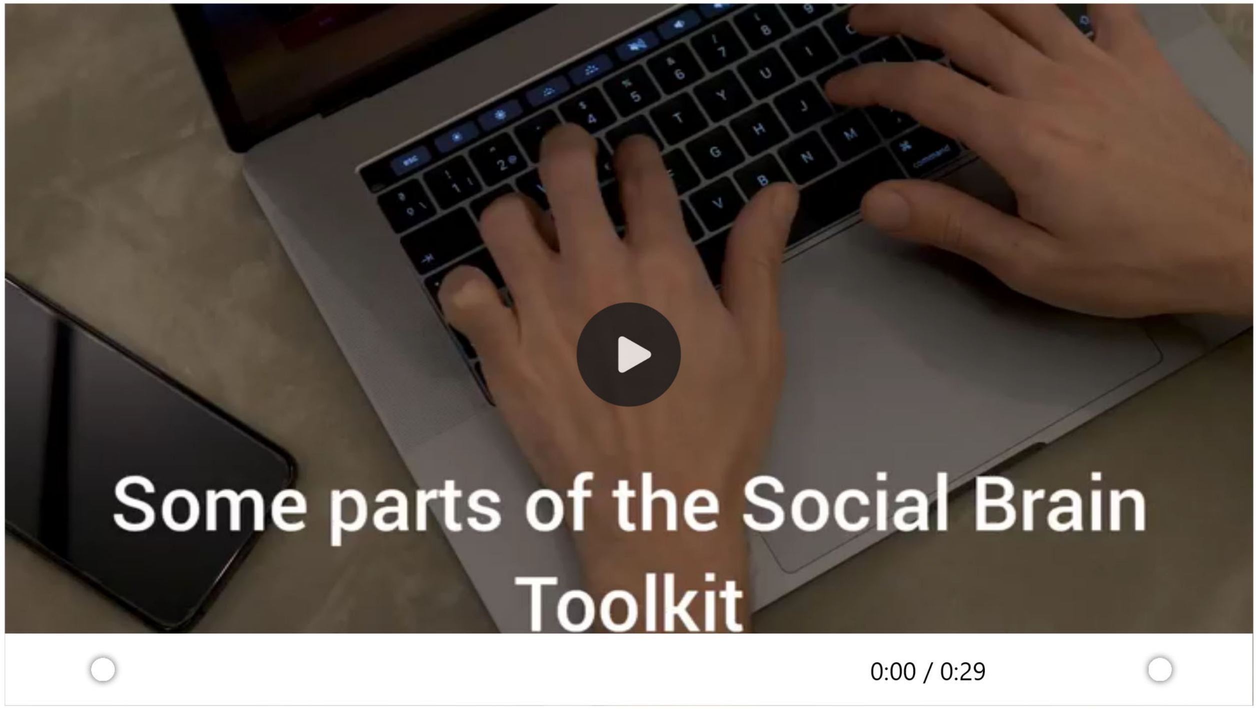

Here is a transcript of the video if you'd like to read it again.

Some parts of the Social Brain Toolkit ask people to work on a device or computer to:

1. *type answers,*
2. *watch videos*

[illegible] and learn online

Other parts ask people to work on a computer to:

- *make video calls*
- *record and upload conversations,*
- *and learn online with a therapist and someone they know*

This **technology** will affect people's use of the Social Brain Toolkit.

- ◯ Strongly agree
- ◯ Somewhat agree
- ◯ Neither agree nor disagree
- ◯ Somewhat disagree
- ◯ Strongly disagree

Why do you think so?

Challenge C: The benefit of the Social Brain Toolkit

Another challenge is **showing the benefit of the Social Brain Toolkit**.

Here is a video explaining some of the reasons why.

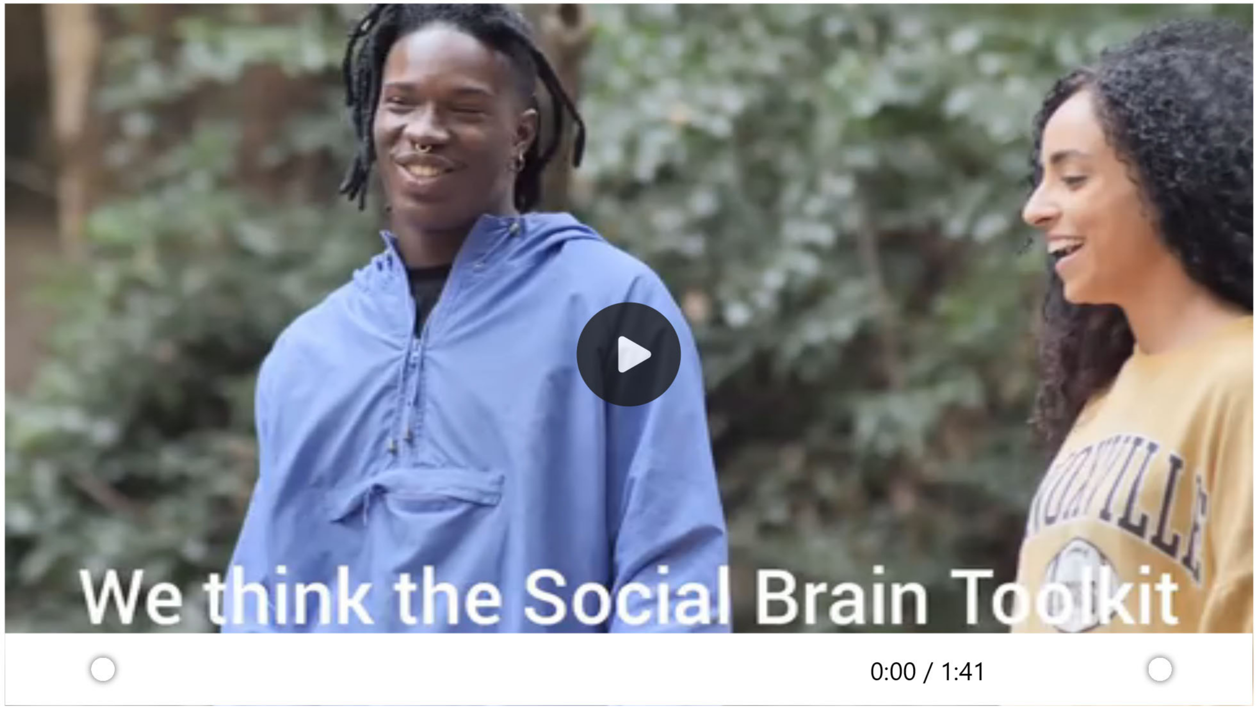

Here is a transcript of the video if you'd like to read it again.

We think the Social Brain Toolkit is valuable because:

- *communication after brain injury is important,*
- *There are many people in the world who need communication training*

The Social Brain Toolkit being online could help more people access communication training.

- *it could help services reach people who can’t come to therapy face-to-face*
- *it helps clinicians follow international guidelines that people with brain injury and their family friends and partners should get training after the injury.*
- *and online treatment can save travel, stress and effort.*

But some people might

- *think it is not worth investing in online care.*
- *worry it takes away therapist jobs,*
- *think it is not as good as face-to-face therapy,*
- *think that is not fair to people without internet or*
- *think it costs too much to set up and keep online services in a clinic.*

So we have to show that the Social Brain Toolkit:

- *helps people,*
- *is not worse than face-to-face care,*
- *helps services reach more people,*
- *is worth the cost*

we don't show this value, the Toolkit could someday disappear without enough money to keep it going.

Being able to show the benefit of the Social Brain Toolkit will affect its use.

- Strongly agree
- Somewhat agree
- Neither agree nor disagree
- Somewhat disagree
- Strongly disagree

Why do you think so?

Challenge D: Changes to routine

Another challenge is **how much change people need to make their routine to use the Social Brain Toolkit**. Here is a video explaining some reasons why:

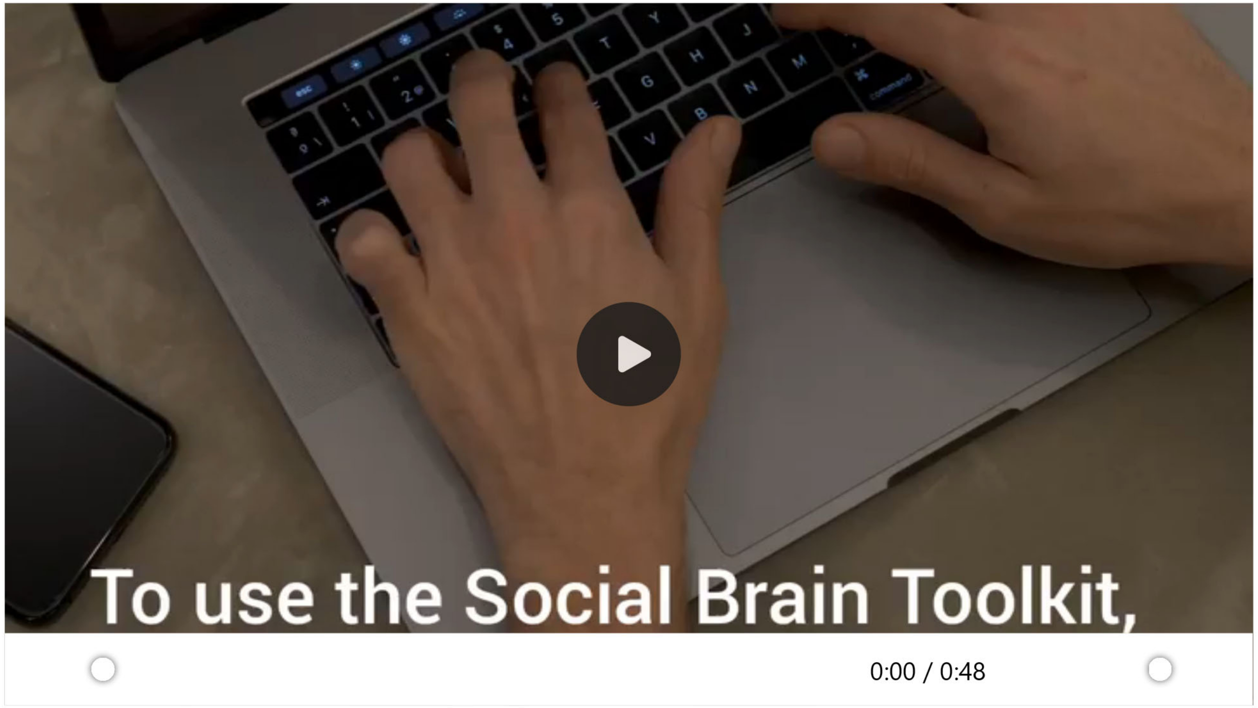

Here is a transcript of the video if you'd like to read it again.

friends, and partners will need to:

· *log in regularly to finish a course*

· *make regular online video calls,*

· *make and upload recordings,*

· *or watch videos and type answers online.*

Clinicians need to be okay with:

· *providing care online*

· *expecting to build a relationship and see progress online,*

· *people managing their own learning instead of making an appointment.*

If these changes are too inconvenient, odd or scary, people might not finish the online training, and clinicians might stick to face-to-face appointments.

How much **change people need to make to their routine** to use the Toolkit will affect people's use of the Social Brain Toolkit.

- ◯ Strongly agree
- ◯ Somewhat agree
- ◯

strongly disagree

Why do you think so?

Challenge E: Services rolling out the Social Brain Toolkit

Another challenge is the rollout of the Toolkit in services. Here is a video of some of the reasons why.

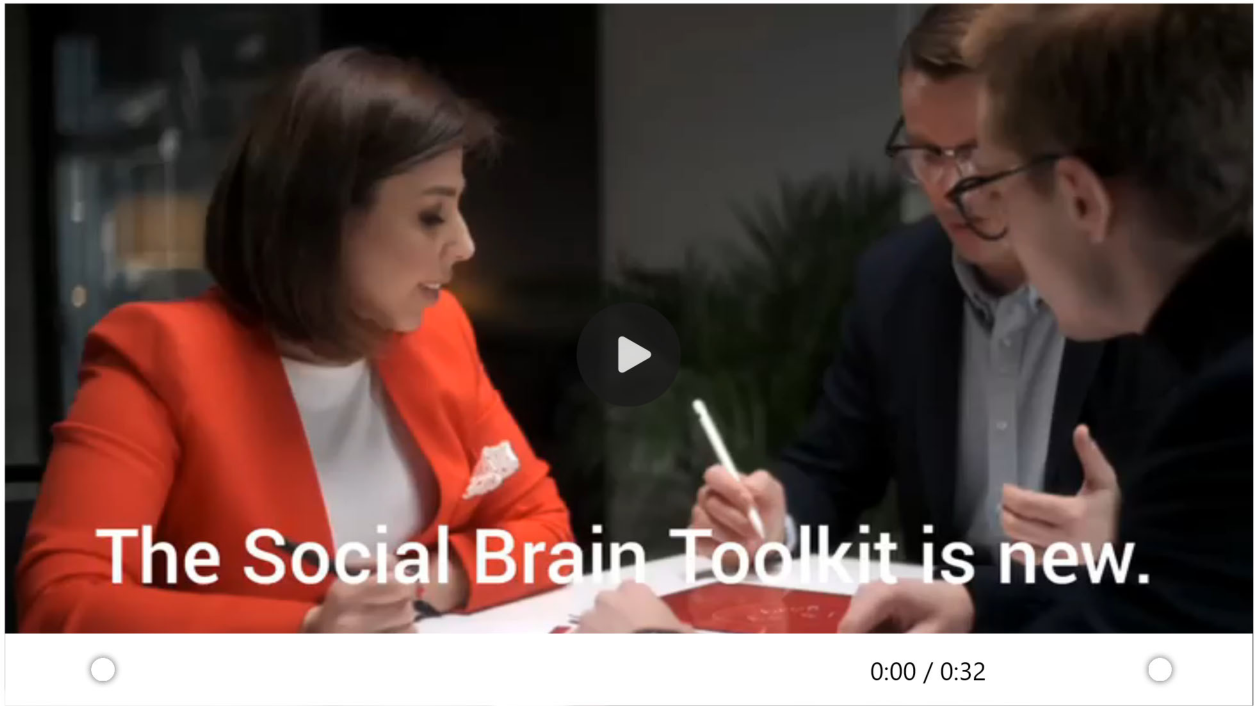

Here is a transcript of the video if you'd like to read it again.

The Social Brain Toolkit is new. Rolling out something new in a health service needs services, managers and therapists to:

- *Put time, effort and money into the change*

· *make sure it lines up with what the service already does or be open to new ideas*

But not all services will be this willing, collaborative, or have enough resources.

A service's **effort to roll out these changes** will affect people's use of the Social Brain Toolkit.

- ◯ Strongly agree
- ◯ Somewhat agree
- ◯ Neither agree nor disagree
- ◯ Somewhat disagree
- ◯ Strongly disagree

Why do you think so?

Challenge F: Society's rules and attitudes towards online healthcare

Another possible challenge is **society's rules and attitudes towards online healthcare**. Here is a video explaining some reasons why.

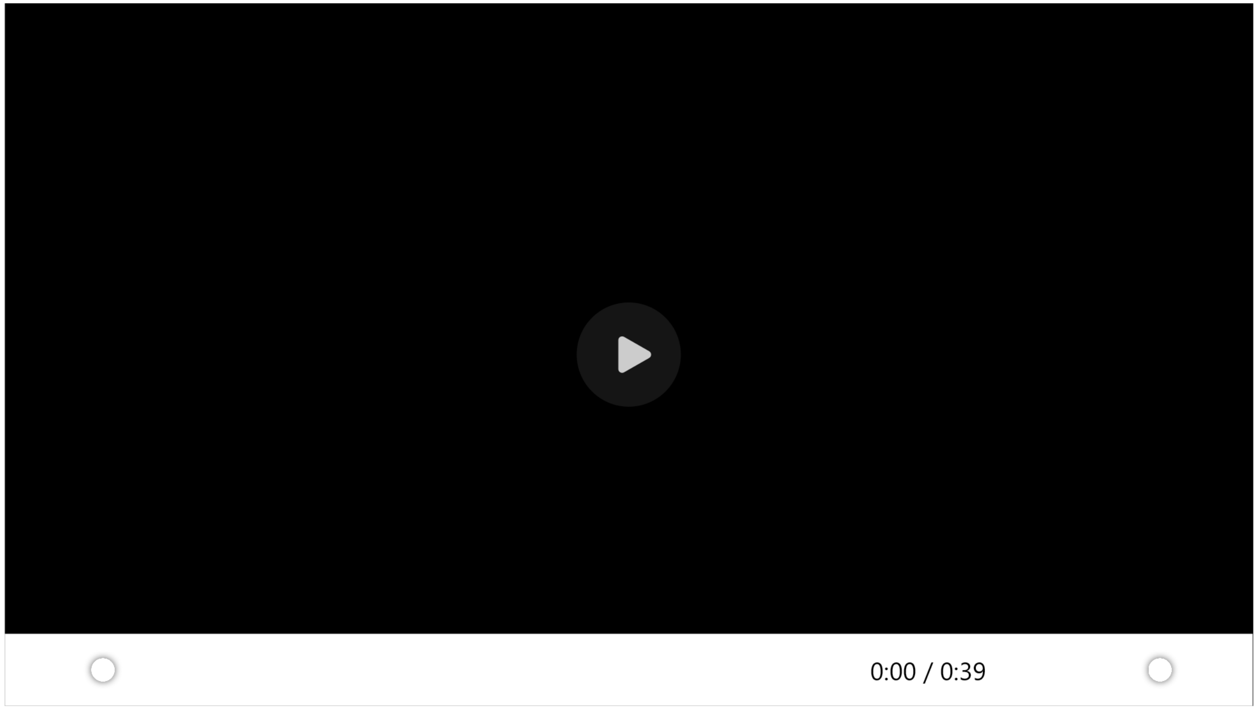

Here is a transcript of the video if you would like to read it.

Rules, laws and attitudes about online healthcare change

· *between states and countries*

· *between political leaders*

· *between types of technology*

· *over time*

People are getting used to using online services and products, and online healthcare, especially since COVID-19.

But some people might

· *think online care is not as good as face-to-face care,*

· *worry online care doesn't have enough rules around quality or privacy*

· *worry online care takes therapists jobs.*

Why do you think so?

Society's rules and attitudes toward online healthcare will affect [illegible]

○ Somewhat disagree

○ Strongly disagree

Challenge G: Problem-solving over time

The seventh challenge is **problem-solving over time**. Here is a video explaining some reasons why.

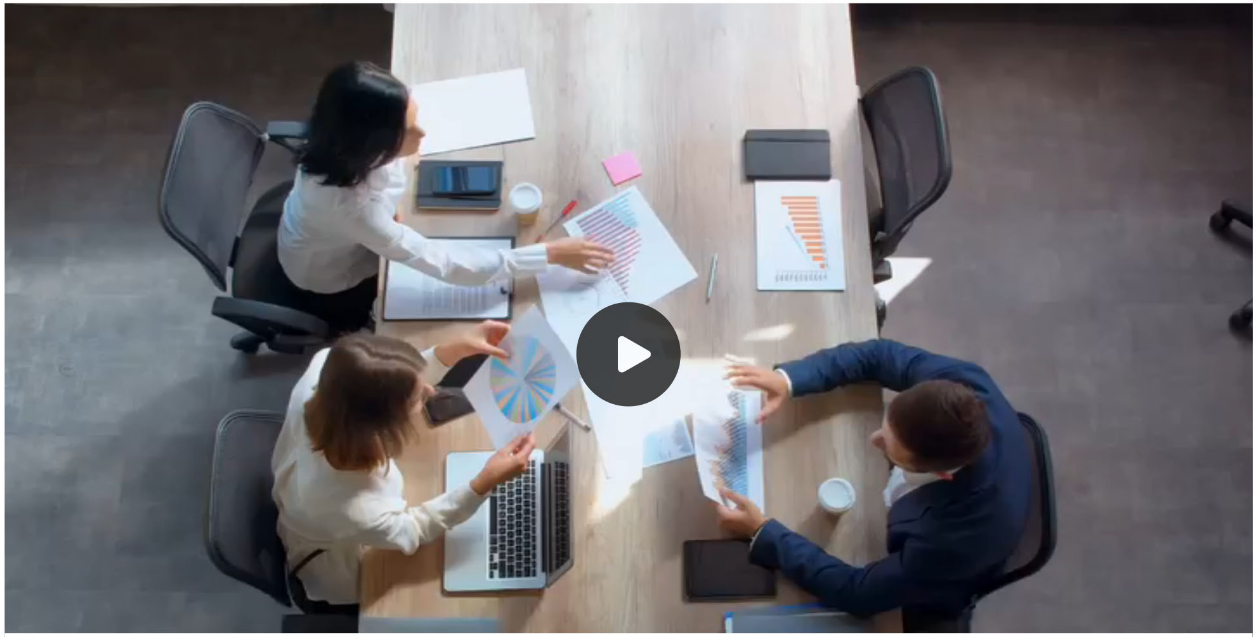

0:00 / 0:36

Here is a transcript of the video if you would like to read it.

Services and therapists will need to keep an eye on how much and how well people are using the Social Brain Toolkit over time.

- *Notice people's problems using or offering the Toolkit*
- *think of ways to fix any problems*
- *and change anything that's not working*

If there are problems, or challenges, will services give up?

Or, will they think of new ways to keep offering the Social Brain Toolkit when technologies, rules and procedures change?

Having to problem-solve over time will affect people's use of the Social Brain Toolkit.

- ○ Strongly agree
- ○ Somewhat agree
- ○ Neither agree nor disagree
- ○ Somewhat disagree
- ○ Strongly disagree

[illegible]

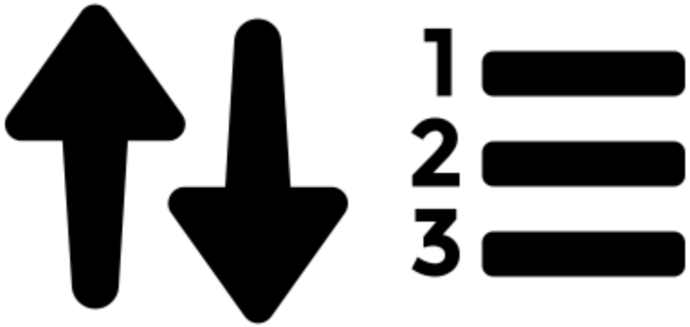

Out of all the challenges, which do you think is the most important? Please move them in order from 1 to 7.

A. Difficulties related to brain injury

B. The technology

C. Showing the value of the Social Brain Toolkit

D. Changes people need to make to use it

E. A service's effort to roll out the treatment

F. Society's rules and attitudes towards online healthcare

G. Problem-solving over time

Please check that the seven are in order.

Can you think of any other challenges, solutions or comments?

- ○ Yes. Please specify below.
- ○ No. Click "Next" to finish the survey.

Powered by Qualtrics
