# Supplementary material for: Coproducing Knowledge of the Implementation of Complex Digital Health Interventions for Adults with Acquired Brain Injury and their Communication Partners: Protocol for a Mixed Methods Study
Source: JMIR Res Protoc. 2022 Jan 10;11(1):e35080. doi: 10.2196/35080 (PMC8787662; doi:10.2196/35080)
Supplement: Multimedia Appendix 10 [file resprot_v11i1e35080_app10.docx]

**Focus group protocol**

| Topic | Time Allocation | Sub-topic | Specific time allocation | Supported Communication |
| --- | --- | --- | --- | --- |
| Welcome | 10 mins | Plenary welcome/briefing/ instructions | 10 minutes | Visually supported slides, check in with participants |
| Introductions | 10 mins | - Introductions within each focus group | 10 minute breakout room | N/A |
| NASS DOMAINS 1 & 2 | 45 mins | - What you/everyone said (Survey results) - What the evidence says (findings from systematic review) | 10 minute plenary presentation | - Visually supported slides presented by speech pathologist researcher - online poll to check understanding of survey and review results - supported communication with speech pathologist in breakout rooms with people with ABI and their carers – clarification of understanding - short, sequentially presented topics to minimise impact of memory difficulties and fatigue |
|  |  | - Discussion | 15 minute breakout room |  |
|  |  | - Plenary feedback | 20 minutes plenary discussion |  |
| BREAK 10 minutes | | | | |
| NASS DOMAIN 4 | 45 mins | - What you/everyone said (Survey results) - What the evidence says (findings from systematic review) | 10 minute plenary presentation | - Visually supported slides presented by speech pathologist researcher - online poll to check understanding of survey and review results - supported communication with speech pathologist in breakout rooms with people with ABI and their carers – clarification of understanding - short, sequentially presented topics to minimise impact of memory difficulties and fatigue |
|  |  | - Discussion | 15 minute breakout room |  |
|  |  | - Plenary feedback | 20 minutes plenary discussion |  |
| BREAK 10 minutes | | | | |
| NASS DOMAIN 3 | 45 mins | - What you/everyone said (Survey results) - What the evidence says (findings from systematic review) | 10 minute plenary presentation | - Visually supported slides presented by speech pathologist researcher - online poll to check understanding of survey and review results - supported communication with speech pathologist in breakout rooms with people with ABI and their carers – clarification of understanding - short, sequentially presented topics to minimise impact of memory difficulties and fatigue |
|  |  | - Discussion | 15 minute breakout room |  |
|  |  | - Plenary feedback | 20 minutes plenary discussion |  |
| Wrap up | 5 minutes | Debriefing/ What happens next? | 5 minutes | Visually supported slides, check in with participants |
